# CRISPR-mediated genome editing of *Plasmodium falciparum* malaria parasites

**DOI:** 10.1186/s13073-014-0063-9

**Published:** 2014-08-26

**Authors:** Marcus CS Lee, David A Fidock

**Affiliations:** Department of Microbiology and Immunology, Columbia University College of Physicians and Surgeons, New York, NY 10032 USA; Division of Infectious Diseases, Department of Medicine, Columbia University College of Physicians and Surgeons, New York, NY 10032 USA

## Abstract

The development of the CRISPR-Cas system is revolutionizing genome editing in a variety of organisms. The system has now been used to manipulate the genome of *Plasmodium falciparum*, the most lethal malaria-causing species. The ability to generate gene deletions or nucleotide substitutions rapidly and economically promises to accelerate the analysis of novel drug targets and to help elucidate the function of specific genes or gene families, while complementing genome-wide association studies.

## Genetic manipulation of the malaria parasite

The global malaria burden exceeds 200 million infections per year, resulting in over 600,000 deaths annually. Most fatalities are attributable to blood-stage infections by the apicomplexan parasite *Plasmodium falciparum*. The current frontline treatments are artemisinin-based combination therapies, whose clinical use is at the scale of hundreds of millions of doses per year. The recent emergence of resistance to artemisinin in South East Asia is of global concern, given the devastating loss of the former frontline therapies chloroquine and pyrimethamine-sulfadoxine to drug-resistant strains of *P. falciparum* [1].

Genetic crosses and genome-wide association studies (GWAS) have been instrumental in uncovering candidate genes that are involved in resistance phenotypes. Nevertheless, confirmation of their actual contribution to resistance requires efficient tools to genetically modify *P. falciparum* parasites cultured *in vitro*. These transgenic lines could also be used to generate insights into the underlying mechanisms of drug action and parasite resistance. Genetic methodologies are also increasingly used to dissect molecular components of *P. falciparum* pathogenesis, including processes of host-cell invasion, protein trafficking inside the infected host cells, virulence, and immune evasion.

Steady progress has been made in transforming *P. falciparum* into a genetically tractable laboratory organism. Important milestones include the establishment of *in vitro* culture systems in 1979, transfection techniques in 1995, and the sequencing of the genome in 2002 [1]. Nevertheless, methods to engineer the *P. falciparum* genome have been hampered by limited tools and inefficiencies in transfection and integration. Furthermore, RNA interference (RNAi) is not effective in *P. falciparum*, unlike mammalian systems.

**Figure 1:**
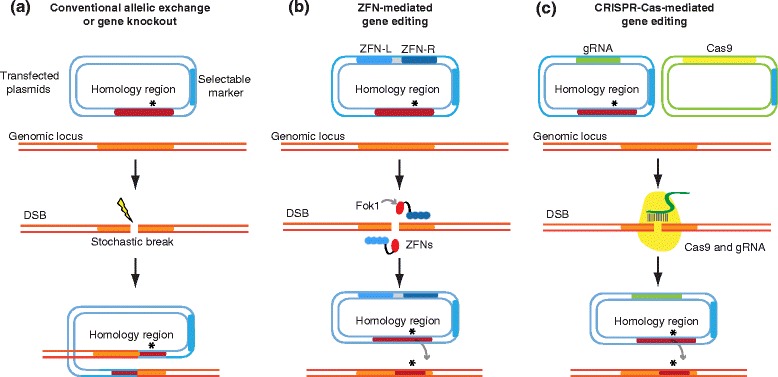
**Genome modification strategies for** ***P. falciparum*** **. (A)** Conventional allelic exchange or knockout strategies rely on a rare homology-driven integration event, probably resulting from a stochastic double-strand break (DSB) near the target site (asterisk). This approach requires several weeks to months of continuous culture, and yields a complex genomic locus after crossover-mediated recombination that includes a selectable marker and duplicated gene fragments. By contrast, genome-editing approaches are driven by a directed DSB event, mediated by site-specific nucleases expressed from transfected plasmids, triggering homology-directed repair from a donor template to yield gene disruptions or nucleotide substitutions (asterisk). **(B)** In ZFN-based editing, heterodimerization of an engineered pair of ZFNs (ZFN-L and ZFN-R) each fused to a split FokI domain (red) yields a functional nuclease that recognizes the specified target site. **(C)** The two-component CRISPR-Cas system consists of a constant nuclease, Cas9, which is directed to the desired location by RNA-DNA base pairing dictated by an expressed gRNA.

Gene disruption in *P. falciparum* traditionally requires one to three months of continuous culture before one can observe a stochastic homologous recombination event that leads to the integration of an episomally maintained plasmid by either a single- or a double-crossover (Figure 1A). Similar recombination-dependent approaches are used for allelic exchange, for example, to validate potential drug-resistance mutations [1]. The inherent inefficiency of this process has precluded any large-scale gene deletion campaigns, with few exceptions such as the heroic effort that was successful in disrupting 53 out of 83 targeted genes that had potential roles in protein export into the host red blood cells (RBCs) [2]. As described below, genome editing techniques based on the RNA-guided CRISPR (clustered regularly interspaced short palindromic repeats)-Cas (CRISPR-associated proteins) system have now been reported for *P. falciparum* [3], providing a powerful new approach that can be used to interrogate the malarial parasite genome.

## Harnessing the power of the double-strand break: genome editing using site-specific nucleases

A double-strand break (DSB) in the chromosome of a cell is a potentially catastrophic event, and as a consequence, organisms have developed mechanisms to deal with this contingency. One emergency measure is non-homologous end joining (NHEJ), which simply stitches together the broken DNA ends in a process that can introduce small insertions or deletions. Alternatively, the cell can use the more precise method of homology-directed repair, using a homologous template to guide the repair [4]. Genome editing harnesses these processes experimentally to disrupt or modify genomic loci in defined ways. The common theme with all genome editing approaches is the use of a site-specific nuclease that cleaves the genome at a defined site, thus triggering a repair event that can be guided to yield outcomes ranging from complete gene deletion to more subtle sequence alterations [5].

Customized zinc-finger nucleases (ZFNs), in which the catalytic domain of the Fok1 nuclease is combined with multiple zinc-finger proteins that specify the DNA-binding site, were the first nucleases to be used widely for genome editing in a variety of organisms, including *P. falciparum*. These nucleases allowed parasites with targeted deletions or allelic replacements to be generated considerably faster than had been possible using conventional methods because ZFN-generated DSB events occur shortly after transfection, rather than weeks to months later [6]. More significantly, perhaps, template-guided repair of the DSB allows for the delivery of as little as a single-base change, an application ideally suited for testing the impact of putative drug resistance mutations (Figure 1B). The recombinant locus that results from ZFN editing contrasts with the complex locus formed by conventional allelic exchange, which often includes the selectable marker, thus precluding its use in downstream applications and a partial duplication of the targeted gene (resulting from crossover-mediated plasmid integration; Figure 1A). ZFN editing is a powerful approach, but the cost and non-trivial nature of designing zinc-finger proteins that bind specific sequences necessitates specialized knowledge and limits the scale at which they can be deployed.

## A new platform for gene editing: CRISPR-Cas

An alternative system for genome editing avoids the ZFN design problem by using RNA-DNA base pairing to guide DNA cleavage. CRISPR-Cas systems function in bacterial adaptive immunity as a defense against viruses or other invasive DNA. The CRISPR sequences, acquired initially from foreign DNA, are arrayed in the host bacterial genome and are transcribed and processed to generate short guide RNAs (gRNAs) that direct the Cas endonuclease to complementary target sites. The ease with which the Cas nuclease can be reprogrammed by supplying alternative gRNAs has led to its exceptionally rapid adoption as a genetic tool in a wide variety of organisms [7].

The first 20 nucleotides at the 5′ end of the gRNA determine specificity by homology to the target site, with an additional sequence requirement in the genome of an -NGG- ‘protospacer adjacent motif’ (PAM) immediately downstream of the target site. To date, the most common use in mammalian systems has been to generate loss-of-function alleles by directed cleavage followed by error-prone NHEJ. This approach has permitted not only the targeting of individual genes but also large-scale genetic screens that employ libraries of over 87,000 distinct gRNAs [8].

## CRISPR-Cas gene editing in *Plasmodium* parasites

A new study by Ghorbal *et al.* [3] published in *Nature Biotechnology* has brought the power of the CRISPR-Cas system to *P. falciparum*. Two distinct applications, gene disruption and single-nucleotide gene editing, were demonstrated, highlighting the versatility of this approach. Parasites with disruptions at two non-essential loci, an integrated green fluorescent protein (GFP) reporter gene and the *kahrp* gene, which encodes a protein exported into the host RBC, were efficiently obtained by insertion of a selectable marker within their coding sequences. Similarly effective were more subtle experiments that modified the sequences of two genes, *orc1* and *kelch13*, which have putative roles in gene silencing and emerging resistance to artemisinin, respectively. Parasites that were altered at the appropriate sites were recovered with very high efficiency, despite there being no direct selection for the modification but only for the transfected plasmids, suggesting that neutral or even deleterious mutations can be generated using this system. As with earlier ZFN-editing experiments [6], the desired alterations were probably enriched within the bulk parasite population by the inclusion of silent mutations in the donor sequence that protected both the donor plasmid and edited genomic locus from cleavage. Furthermore, for reasons explained below, the ability to place the DSB site in very close proximity to the desired modification is another driver of efficient editing.

A two-plasmid approach was used by Ghorbal *et al.* [3] to express both the Cas9 nuclease and the gRNA (Figure 1C). Because of the requirement for a precise transcriptional start site for the gRNA, expression in mammalian cells has typically utilized the promoter of the U6 small nuclear RNA (snRNA) component of the spliceosome. U6 snRNA transcription is dependent on RNA polymerase III, which precisely transcribes a variety of non-protein-coding RNAs. Transcription of the U6 product initiates with a guanosine nucleotide, placing some constraint on the selection of target sequences in the genome, which optimally consist of the motif G-N_19_-NGG, with the final three nucleotides (the PAM sequence) critical for cleavage. One notable finding of the Ghorbal *et al.* study [3] is that the use of the *P. falciparum* U6 snRNA upstream regulatory region was able to drive expression of gRNAs in the parasite, but apparently without the functional requirement for the initial guanosine nucleotide. This is significant because it greatly expands the potential target sites within the parasite genome to any sequence with the -NGG PAM motif, a particularly liberating feature for an organism that has an average GC content of only 19.4%.

One quirk of *Plasmodium* biology is that these parasites appear to lack the machinery for NHEJ [6]. Thus, the most basic and scaleable application of the CRISPR-Cas system, cleavage followed by error-prone repair to generate frameshift mutations, is not available to malaria researchers. Nevertheless, an enticing array of other possibilities awaits the field. The ability to express two or more gRNAs within the same cell may permit multiplex cleavage events that can generate large deletions, which could be used to investigate genome duplications that are often associated with drug resistance. In mammalian systems, a nuclease-dead version of Cas9, guided to a target gene, has been shown to act as a roadblock to transcription. Conversely, fusion of this inactive Cas9 to a transcriptional activation domain can increase gene expression [9]. Development of these gene regulatory approaches for *Plasmodium* would more than fill the gap left by the absence of RNAi tools for the parasite.

Robust, inexpensive methods to manipulate the parasite genome efficiently should now accelerate the discovery of new drug targets, the validation of GWAS data from field and clinical samples, and the identification of genes that are important for the critical parasite transitions between the human and mosquito hosts. The best new technologies offer not just improvements to existing methods but transform the very nature of the questions we can ask. The malaria parasite has joined the CRISPR-Cas revolution, and as these tools are brought to bear on a pathogen that is both deadly and biologically fascinating, we eagerly await the insights that are sure to follow.

### Note added in proof

A recent report by Zhang *et al*. [10] describes the use of CRISPR/Cas systems to modify the genome of the rodent malaria parasite, *Plasmodium yoelii*.

## Abbreviations

Cas: CRISPR-associated proteins
CRISPR: clustered regularly interspaced short palindromic repeats
DSB: double-strand break
GFP: green fluorescent protein
gRNA: guide RNA
GWAS: genome-wide association studies
NHEJ: non-homologous end joining
PAM: protospacer adjacent motif
RBC: red blood cell
RNAi: RNA interference
snRNA: small nuclear RNA
ZFN: zinc-finger nuclease
